# A systematic study of the controlled generation of crystalline iron oxide nanoparticles on graphene using a chemical etching process

**DOI:** 10.3762/bjnano.8.202

**Published:** 2017-09-26

**Authors:** Peter Krauß, Jörg Engstler, Jörg J Schneider

**Affiliations:** 1 Fachbereich Chemie, Eduard-Zintl-Institut für Anorganische und Physikalische Chemie, Technische Universität Darmstadt, Alarich-Weiss Str. 12, 64287 Darmstadt, Germany

**Keywords:** carbon nanotubes, chemical vapor deposition, graphene, iron oxide, nanoparticles

## Abstract

Chemical vapor deposition (CVD) of carbon precursors employing a metal catalyst is a well-established method for synthesizing high-quality single-layer graphene. Yet the main challenge of the CVD process is the required transfer of a graphene layer from the substrate surface onto a chosen target substrate. This process is delicate and can severely degrade the quality of the transferred graphene. The protective polymer coatings typically used generate residues and contamination on the ultrathin graphene layer. In this work, we have developed a graphene transfer process which works without a coating and allows the transfer of graphene onto arbitrary substrates without the need for any additional post-processing. During the course of our transfer studies, we found that the etching process that is usually employed can lead to contamination of the graphene layer with the Faradaic etchant component FeCl_3_, resulting in the deposition of iron oxide Fe*_x_*O*_y_* nanoparticles on the graphene surface. We systematically analyzed the removal of the copper substrate layer and verified that crystalline iron oxide nanoparticles could be generated in controllable density on the graphene surface when this process is optimized. It was further confirmed that the Fe*_x_*O*_y_* particles on graphene are active in the catalytic growth of carbon nanotubes when employing a water-assisted CVD process.

## Introduction

Graphene was first described by Boehm and coworkers in the early 1960s [1–4] and later demonstrated by Geim and Novoselov in 2004 using sophisticated and skillfull mechanical exfoliation of highly oriented pyrolytic graphite (HOPG) [5]. This seminal discovery enabled the research field of two-dimensional materials on a broader scope, leading to the dissemination of several top-down and bottom-up approaches to synthesize and isolate graphene, each having their own advantages and disadvantages [5–12]. The most common route to synthesize continuous, large-area graphene is chemical vapor deposition (CVD) using a carbon precursor on a planar metal catalyst substrate [11–13]. A major challenge towards the usage of graphene is the isolation from this planar metal substrate [14–16]. Besides mechanical exfoliation [17–18] and electrochemical delamination [19–21], polymer-supported etching of the substrate is generally used to transfer CVD graphene onto various substrates [14,22–24].

The transfer of CVD graphene by chemical etching is based on the redox reaction between the metal catalyst and an oxidizing agent in aqueous solution [22–23,25–28]. As the metal dissolves in the etchant solution, graphene remains floating on the surface of the solution and can be scooped up with a target substrate. However, chemical etching of graphene often results in tearing of the continuous thin layer by fluid dynamics of the etchant solution, in particular during cleaning where the etchant solution is continously replaced by water. An additional polymer coating is often deposited on the monolayer prior to the etching process to provide mechanical support for the graphene, preventing it from tearing and ripping [14,22–24,29–31]. Most common are thin layers of poly(methyl methacrylate) (PMMA) which can be easily deposited by spin coating from solution [23,25]. The main disadvantage of applying these supporting polymer layers to graphene is their removal during the final steps of the transfer procedure. Dissolving the polymer, for example by using hot acetone, typically leaves residue on the graphene monolayer [22,32–34]. As graphene is close to or even atomically thin, even very minor amounts of contamination can affect its electronic properties [22,33–34]. Additional processing such as thermal annealing in vacuum or hydrogen atmosphere is required to completely remove the protective layer, making polymer-supported graphene transfer laborious and time consuming [35–37].

The standard catalyst and growth substrate used to synthesize graphene by chemical vapor deposition is copper due its effective decomposition of hydrocarbons and its low carbon solubility as compared to the use of nickel as a substrate [12–13,30,38]. For transferring the graphene layer, the copper substrate is dissolved in a Faradaic reaction with aqueous etchants such as iron(III) chloride [25,28], iron(III) nitrate [22–23] or ammonium persulfate [26–27]. Though all of these etchants are suitable oxidizing agents for disolving copper, a clean transfer of graphene, in addition to the polymer transfer, can also be hindered by reactions of the dissolved ions with the graphene surface. Indeed, Alemán et al. reported on the contamination of graphene with iron oxide nanoparticles using iron(III) chloride during the etching process of the copper growth substrate [39]. While this process may be unwanted, it could on the other hand allow for the generation of nanoparticles on the graphene surface under direct, yet controlled conditions. Therefore, we have systematically studied herein the Faradaic etching process in more detail and analyzed different parameters determining the etching outcome. Interestingly we could pinpoint conditions which allow control of the iron oxide particle formation on graphene. With that, we were able to devise a method for graphene/metal oxide composite materials by controlling the Faradaic etching process and could show that the iron oxide particles on the graphene surface are catalytically active in the growth of carbon nanotubes on the graphene surface. In order to realize a systematic investigation of that effect, we have developed a modified chemical etching process in which the protective polymer coating on the as-synthesized graphene is replaced by a protective frame which does not cover the graphene but which allows the detached graphene layer to be handled in a secure way. In addition, subdividing the etching chamber in two isolated yet connected compartments helps to further reduce mechanical stress on the graphene surface. Thus, etching and washing of the graphene occurs in a controlled environment, preventing mechanical agitation of the ultrathin graphene layer. As there is no direct contact between the graphene and the protective frame, any contamination with carbon residue can be ruled out, thus replacing the need for further post-processing of graphene.

## Results and Discussion

**Figure 1:**
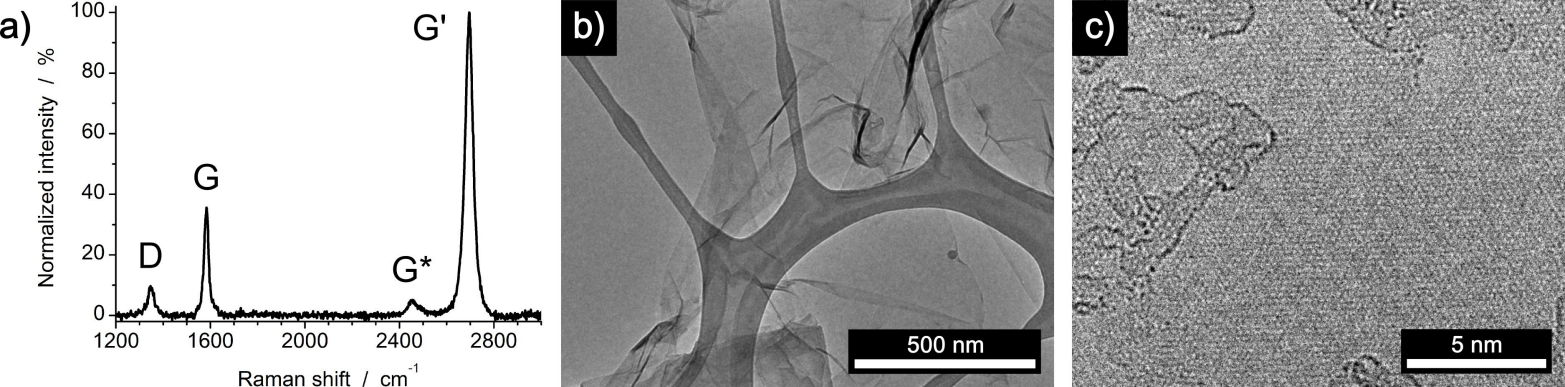
Characterization of graphene obtained by the modified etching process with an aqueous solution of ammonium persulfate. a) Representative Raman spectrum of graphene normalized to the intensity of the G’ peak. The spectral location of the G and G’ peak, the intensity ratio of G’/G and the full width at half maximum of the G’ peak indicate the synthesis of single to few layer graphene. b) and c) High-resolution TEM images of as-synthesized graphene.

Graphene was synthesized by CVD on a copper substrate using methane. For the copper substrate etching process used to isolate the graphene, we used an encircling polymer frame made of standard adhesive tape (e.g., tesa tape from Tesa SE), which protects the exposed edges of the synthesized graphene layer on the copper substrate (see Supporting Information File 1, Figure S1). These are the sites where mechanical agitation by fluid flow of the etching liquid is strongest and often results in tearing and ripping of the detached graphene layer. A compartmentalized etching chamber drastically reduces the strain on the graphene during this procedure. This is due to the fact that the most mechanically demanding step that occurs during the isolation of the graphene layer (i.e., the cleaning process which entails subsequent washing steps to eliminate residue from the etchant) is carried out in an isolated compartment. Carefully draining the etchant from and refilling water into the isolated outer compartment allows the etchant solvent to drain by diffusive processes. This minimizes the horizontal movement of the uncoated, fragile carbon layer while it is located in the inner compartment. Breakage of the layer may eventually still occur, especially during the critical step of scooping up the floating graphene with a target substrate, yet continuous graphene layers can be transferred. As the graphene itself was not coated by a polymer layer, additional post-processing steps to remove residue after the transfer are not required. A detailed schematic of the modified etching process, which is the basis of the following systematic study, is shown in Supporting Information File 1, Figure S2. Employing the developed, modified etching process using aqueous ammonium persulfate as an etchant [26–27], graphene was transferred onto (i) a SiO_2_/Si wafer and (ii) a transmission electron microscopy (TEM) grid and characterized using Raman spectroscopy and high-resolution TEM (Figure 1).

The Raman spectrum shows the characteristic D band peak at 1348 cm^−1^, a G peak at 1584 cm^−1^, the G* peak at 2450 cm^−1^ and the G’ peak at 2696 cm^−1^. The position of the peaks, the peak habitus, the full width at half maximum of the G’ peak and the intensity ratio of the G’ to G peaks for different spots for different samples indicate the viability of the developed transfer method [40–44]. It is thus demonstrated that single to few layer graphene can be obtained with an average of one to three layers in a highly reproducable manner. High-resolution TEM images of the transferred graphene nicely confirm the results of the Raman analysis. Aside from sparse amorphous contamination, the obtained graphene is free of any debris and the copper substrate is completely removed by the newly developed etching process.

**Figure 2:**
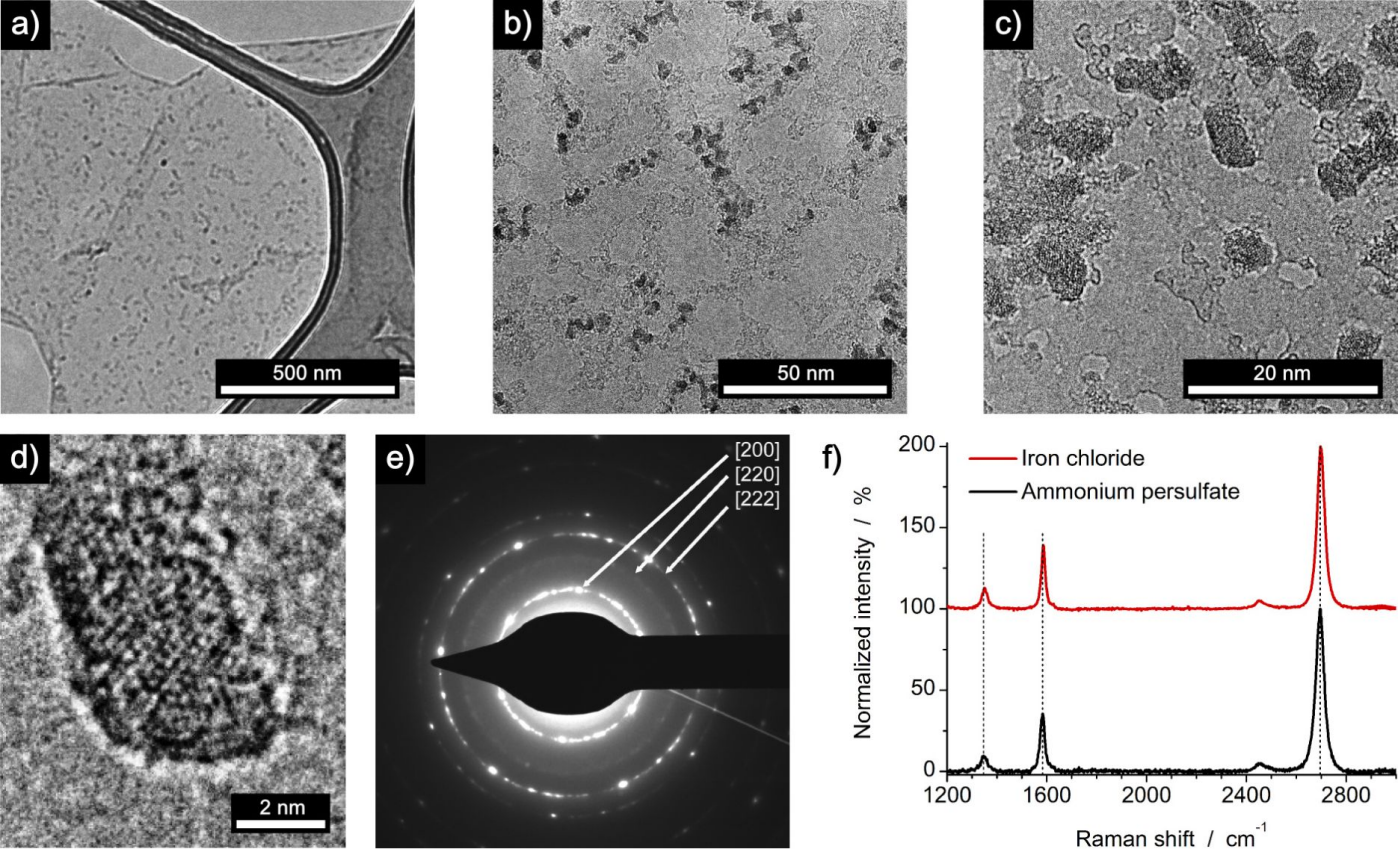
Characterization of CVD graphene transferred onto a TEM grid and a SiO_2_/Si wafer by the modified etching process. The etchant was 1 M iron(III) chloride in 10% hydrochloric acid solution. a–d) TEM micrographs of graphene with deposited crystalline iron oxide nanoparticles. e) SAED of cubic iron oxide Fe_0.942_O nanoparticles on graphene. f) Raman spectra of graphene after the transfer process with an aqueous solution of ammonium persulfate (black trace) and iron(III) chloride (red trace). Spectra are normalized to the intensity of the G’ peak and show similar peak positions, shapes and intensities.

Next, we studied the use of a hydrochloric solution of iron(III) chloride as an etchant for graphene transfer in our modified etching process. Indeed we could confirm the contamination of synthesized and transferred graphene with crystalline iron oxide nanoparticles (Figure 2), as was first reported by Alemán et al. [39]. Selected area electron diffraction (SAED) of the detected nanoparticles indicated formation of cubic iron oxide of composition Fe_0.942_O (Figure 2e) which was further proven by energy-dispersive X-ray (EDX) spectroscopy (Figure S3, Supporting Information File 1). The formed iron oxide nanoparticles with an average diameter of 3 ± 1 nm are highly crystalline and randomly distributed on the graphene surface. Raman spectroscopy of the iron oxide nanoparticles on the graphene composite exhibits a spectrum nearly identical to the peak positions and intensities of the samples which were etched with ammonium persulfate, prohibiting an unequivocal analysis concerning iron oxide deposition (Figure 2f).

**Figure 3:**
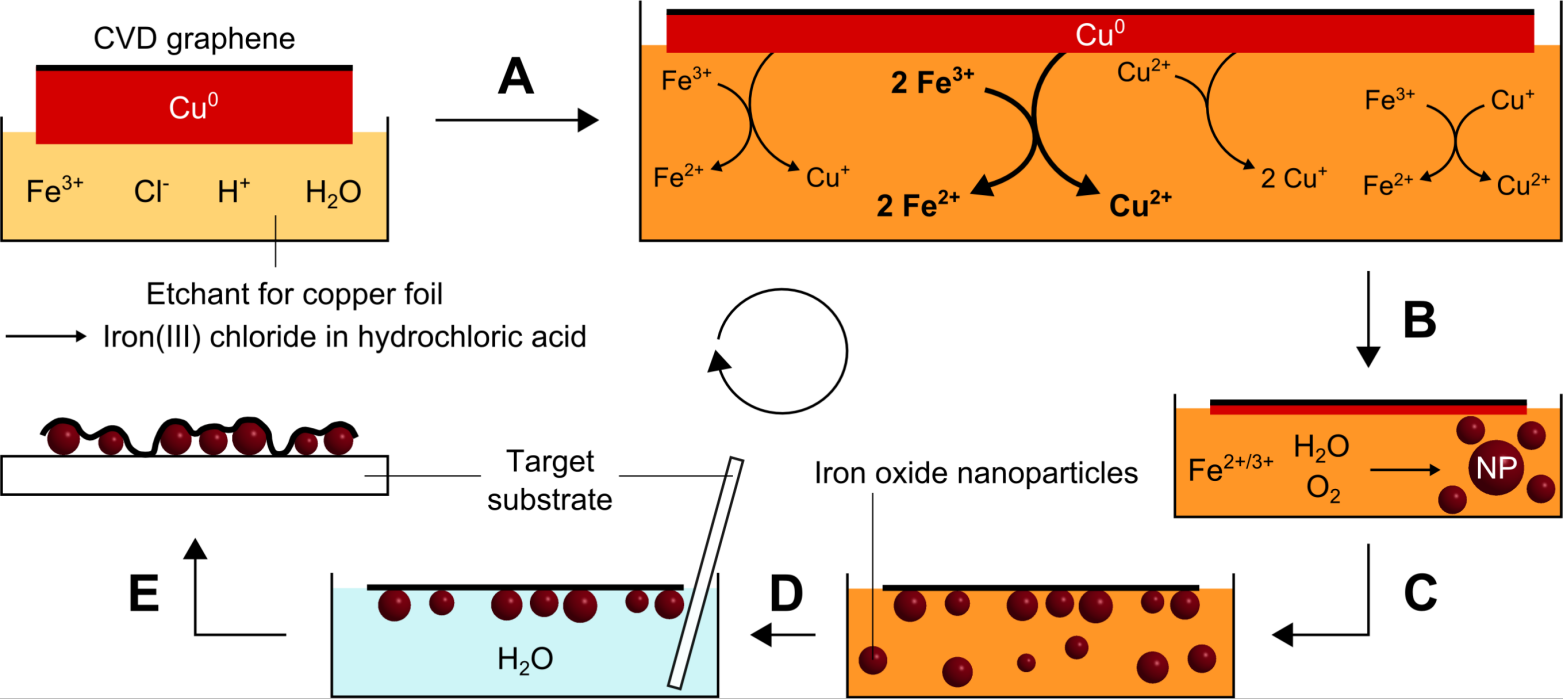
Schematic of the proposed mechanism for the formation of iron oxide nanoparticles on graphene during the modified transfer of graphene by chemical etching. (A) Etching of copper using a hydrochloric solution of iron(III) chloride. (B) Formation of iron oxide nanoparticles. (C) Adsorption of nanoparticles on CVD graphene. (D) Cleaning of graphene and removal of free nanoparticles by dilution with water. (E) Transfer of the as-functionalized CVD graphene onto a target substrate.

A potential mechanism for the formation of iron(II) oxide nanoparticles on graphene during the Faradaic etching process is depicted in Figure 3. As the substrate, elemental copper, is oxidized, iron(III) ions are reduced to iron(II) ions. A reaction between these iron(II) ions and oxygen and/or water should result in the formation of non-stoichiometric iron(II) oxide nanoparticles. After successfully transferring the functionalized CVD graphene, iron(II) oxide nanoparticles are located between the target substrate and the carbon monolayer.

**Figure 4:**
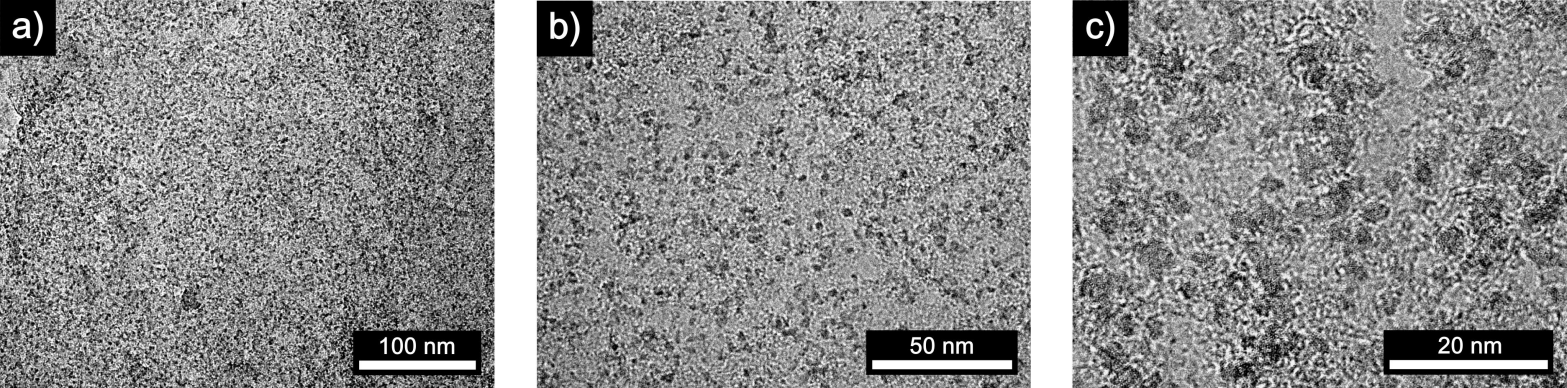
TEM micrographs of graphene functionalized with crystalline iron oxide nanoparticles using an increased ratio of copper to iron oxide etchant. Magnification increases from a) to c). Graphene on copper foil was etched with a solution of 1 M iron(III) chloride in 10% hydrochloric acid.

It is interesting to note, that the as-synthesized iron(II) oxide nanoparticles preferentially adsorb on graphene sites where multiple layers occur (Figure S4, Supporting Information File 1). These areas may provide additional binding sites for the nanoparticles since they act like edge structures on an atomically flat, defect-free, continuous graphene monolayer. To understand the process in more detail we further investigated individual parameters of the Faradaic etching process with iron oxide nanoparticles. Consequently, the ratio of copper substrate to etchant solution, the concentration of the latter iron(III) chloride concentration, as well as the weight percent of hydrochloric acid and the etching time, were all varied. To study the dependence of iron oxide particle formation vs chemical etching of copper with iron(III) chloride, varying ratios of copper to etchant were studied first. Nine copper substrates of identical size were pre-etched with iron(III) chloride followed by graphene growth via CVD and final complete substrate etching to isolate the synthesized graphene using the as-prepared Fe(III) containing etchant. This process increases the number of observed iron(II) oxide nanoparticles on the graphene drastically as can be seen in Figure 4. As additional iron(II) oxide particle formation by pre-chemical etching is thus confirmed, the number of iron(II) oxide nanoparticles can be customized in this process by adapting the ratio of copper substrate to iron(III) etchant employed.

We also investigated the formation of iron oxide nanoparticles as a function of the concentration of the iron(III) chloride etchant by reducing its concentration to 0.5 M and 0.25 M in 10% hydrochloric acid. While using a concentration of 0.5 M iron(III) chloride does not show any significant variation in number, size or shape of the formed iron oxide nanoparticles (see Supporting Information File 1, Figure S5a and Figure S5b), further reduction to 0.25 M alters the size of the iron oxide particles deposited significantly down to 2 ± 1 nm on average (see Supporting Information File 1, Figure S5c and Figure S5d).

When a solution of 1 M iron(III) chloride in 10%, 20%, 30% and 37% hydrochloric acid was used for 3 h for etching, the density of iron oxide nanoparticles deposited on graphene is drastically reduced. Using a concentrated hydrochloric acid (37%) as the solvent for the iron chloride etchant indeed reduces the number of formed oxide particles to a minimum (Figure S6, Supporting Information File 1). These combined results prove a correlation between the weight percent of hydrochloric acid and the nucleation rate of iron oxide nanoparticles and indicates a particle formation involving decomposition of intermediate iron(II) hydroxide formed in the etching process. Increasing the weight percentage of HCl favors the back reaction and thus prevents further particle growth according to: FeCl_2_ + 2 H_2_O 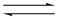 Fe(OH)_2_ + 2 HCl.

We also investigated the influence of the etching time. Samples were etched with a hydrochloric solution containing 1 M iron(III) chloride for different periods of time. The etching process was terminated by quenching the etchant with water after periods of 15 min to 24 h (Figure S7, Supporting Information File 1). As the copper substrate has to be completely etched for TEM analysis, a period of 15 min is the minimum time for the visual removal of the copper substrate. However, no significant variation in number, size or shape of the iron oxide nanoparticles can be observed for the different time periods of etching.

**Figure 5:**
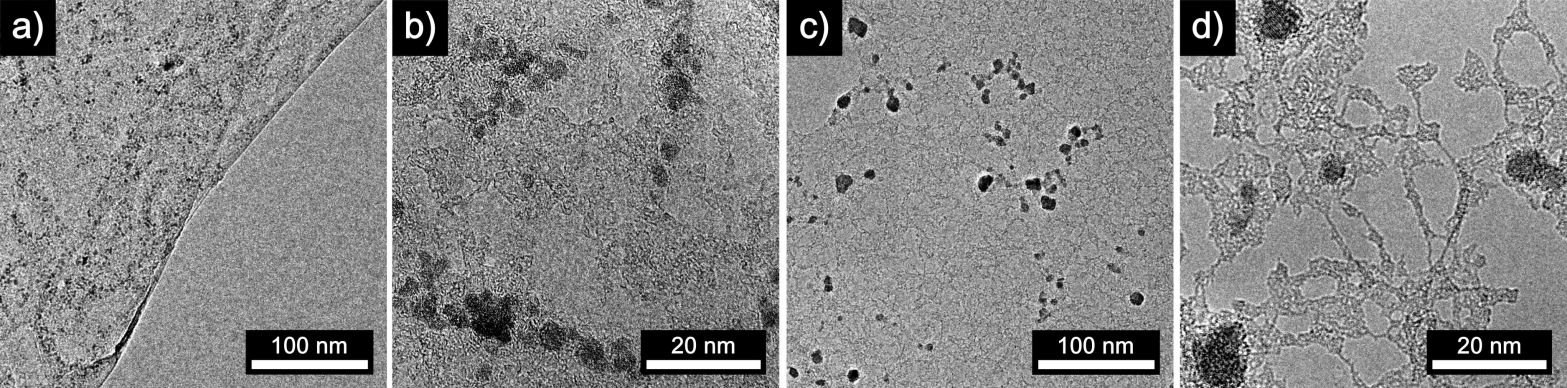
TEM micrographs of iron oxide nanoparticles on graphene; a,b) before and c,d) after thermal annealing at 450 °C for 24 h in a hydrogen atmosphere. The nanoparticles are highly mobile and tend to agglomerate during the heat treatment procedure.

Post-processing by annealing at elevated temperature in a hydrogen atmosphere reveals a high mobility of the iron oxide nanoparticles on graphene. After heat treatment of the graphene samples which were transferred onto TEM grids (for 24 h at 450 °C), the iron oxide nanoparticles (which are initially in the subnanometer to nanometer range and could only barely be detected) agglomerated significantly due to Ostwald ripening and were found to have diameters in the range of 9 ± 4 nm after this annealing procedure (Figure 5).

**Figure 6:**
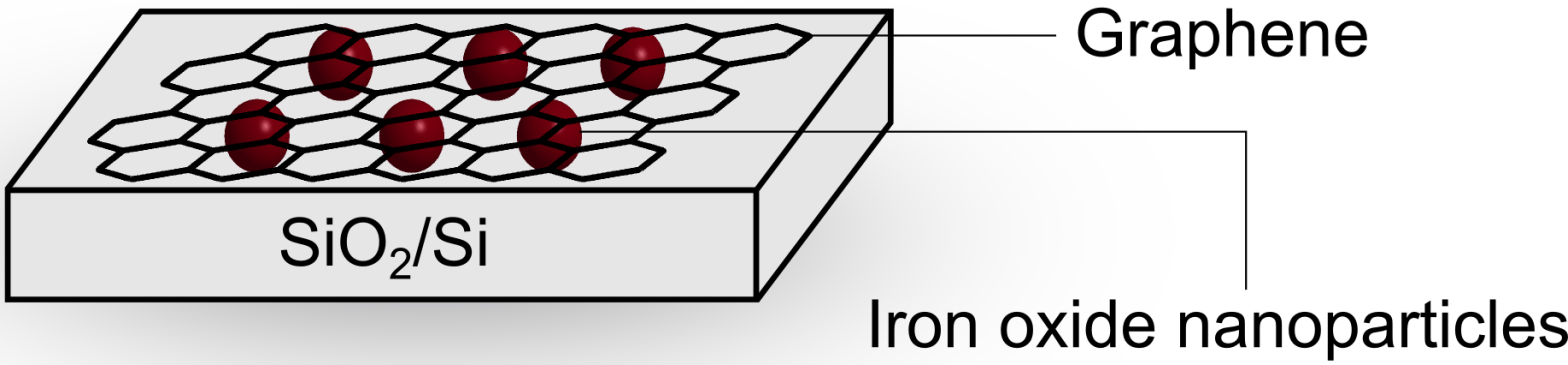
Iron oxide decorated graphene layer on a SiO_2_/Si wafer for CNT growth. The nanoparticles are located between the substrate and the CVD graphene. For a schematic of the complete growth process see Figure 8.

To summarize, functionalization of CVD-derived graphene with iron oxide nanoparticles is possible using customized etchants and/or additional processing techniques. The number of iron oxide particles in this process can be adjusted by increasing the ratio of copper to iron chloride etchant and by adapting the weight percent of hydrochloric acid. The concentration of iron(III) chloride and heat treatment of the samples affects the morphology of the nanoparticles. In addition, we have studied the as-prepared iron oxide nanoparticles on graphene composite material for direct synthesis of carbon nanotubes (CNTs) under CVD conditions. The graphene was transferred onto a SiO_2_/Si wafer after CVD synthesis on a copper substrate (Figure 6). The copper substrate was etched with a solution of 1 M iron(III) chloride in 10% hydrochloric acid. The ratio of copper to etchant was increased as described previously in order to obtain a higher number of iron oxide nanoparticles which serve as a catalyst for CNT growth. The water-assisted CVD growth of CNTs was then performed at 875 °C in an argon/hydrogen atmosphere [45–47].

**Figure 7:**
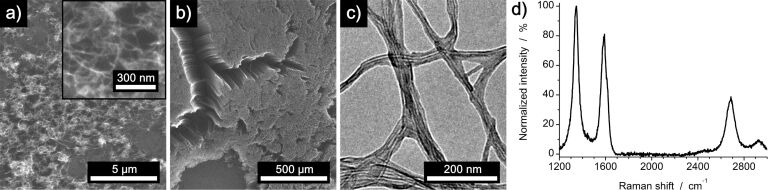
Characterization of CNTs grown on iron oxide nanoparticles/graphene obtained by a modified copper etching process using a solution of 1 M iron(III) chloride in 10% hydrochloric acid and an increased ratio of copper to FeCl_3_ etchant. a) SEM images of substrate areas where randomly oriented growth of CNTs was observed (inset: higher magnification image). b) SEM image of an area with VACNTs. c) TEM and d) Raman spectroscopy of VACNTs confirms the synthesis of multiwalled carbon nanotubes with an average number of 4–7 walls.

Analysis by scanning electron microscopy (SEM) confirms the growth of CNTs on metal oxide decorated graphene samples. The CNTs have an outer diameter of about 10–20 nm (Figure 7a). In selected regions of the graphene substrate, growth of vertically aligned CNTs (VACNTs) was observed (Figure 7b). Analysis of these VACNTs by TEM and Raman spectroscopy (Figure 7c,d) proves the synthesis of multiwalled carbon nanotubes with an average number of 4–7 walls. Using graphene which was functionalized with iron oxide nanoparticles of lower density (comparable to Figure 2) results in a less dense growth of CNTs, yet random areas of VACNTs still exist under these growth conditions (Figure S8, Supporting Information File 1). We speculate that in areas where VACNT growth occurs, the catalyst particle size and density are in the range that a dense growth, supported by van der Waals interactions between growing neighboring CNTs, can occur.

**Figure 8:**
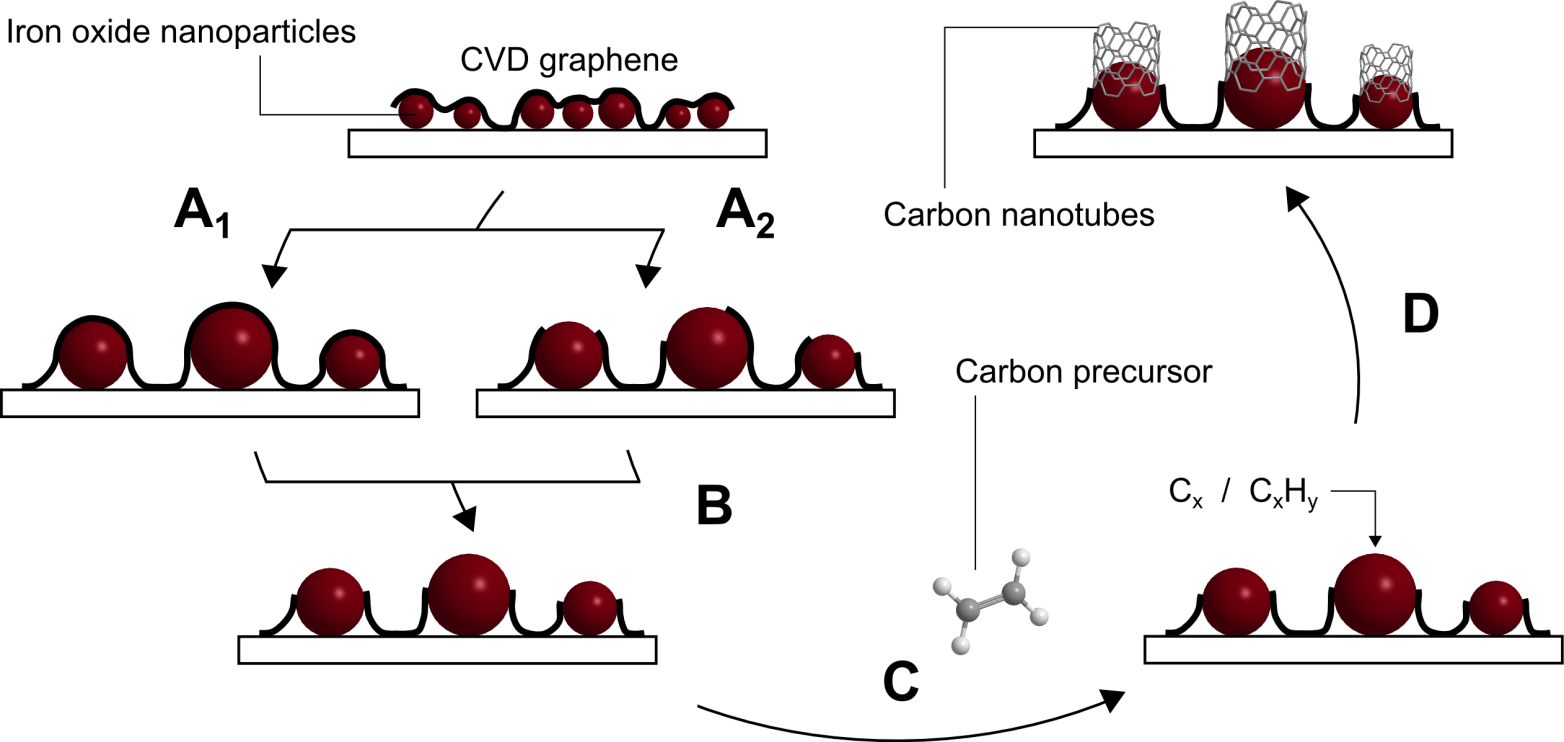
Proposed mechanism for the synthesis of CNTs on a metal oxide decorated graphene surface. (A) As the temperature increases during the CVD process, the iron oxide nanoparticles tend to agglomerate. The graphene can either remain intact (A_1_) or may rupture during this process (A_2_). (B) Graphene on the nanoparticle surface may rupture and/or decompose on the iron oxide nanoparticle surface at higher temperatures. (C) Synthesis of CNTs is initiated by exposure of the iron oxide nanoparticle surface to the carbon precursor. (D) Growth of CNTs on top of the graphene. Note that the metal oxide particles are found underneath the graphene layer as the etchant solution agitates from below.

A schematic formation mechanism for the growth of CNTs on iron oxide nanoparticles/graphene is shown in Figure 8. As the temperature increases, the iron oxide nanoparticles agglomerate, resulting in a diameter increase. Due to mechanical stress, graphene on top of the particles may rupture during this process. In addition, even a local decomposition of the graphene layer on the nanoparticles is possible at higher temperatures. Finally, the nanoparticles are exposed to the carbon precursor and the CNTs grow on top of the graphene. Heating in a hydrogen-containing atmosphere may reduce the iron oxide nanoparticles, yet previous results under harsher conditions (higher temperature, longer time of hydrogen treatment) indicated that only a small percentage of iron oxide is actually reduced under such conditions [48].

We also investigated the growth of CNTs on iron oxide nanoparticles/graphene without introducing a carbon precursor during water-assisted CVD in order to see whether CNT growth is due to the carbon precursor employed. Therefore, apart from ethene gas flow, which was substituted by argon, all parameters for CNT synthesis were unchanged. As no growth can be detected, the results confirm that the synthesis of carbon nanotubes on functionalized graphene is due to the decomposition of the gaseous carbon precursor and does not come from the graphene itself as a possible carbon source. In addition, samples without iron oxide nanoparticles show no growth of CNTs when using ethene as a growth component, confirming their catalytic activity.

## Conclusion

We introduced a modified solution-based etching process for CVD graphene by replacing the regular protective polymer coating with an encircling polymer frame surrounding graphene and by subdividing the etching chamber into two connected individual compartments. Using this modified chemical etching process, an additional post-processing to remove polymer residues from graphene is not required. We further investigated functionalization of graphene with iron oxide nanoparticles by using hydrochloric solutions of iron(III) chloride as the etchant in the modified transfer process. A controlled functionalization with iron(II) oxide nanoparticles is possible by adjusting the ratio of copper to etchant, the concentration of iron(III) chloride and weight percent of hydrochloric acid employed. The annealing of functionalized graphene in a hydrogen atmosphere resulted in agglomeration of the iron(II) oxide nanoparticles, increasing their average diameter from 3 nm to 9 nm. The synthesis of multiwalled carbon nanotubes on iron oxide nanoparticle/graphene composites was demonstrated. This resulted in irregular CNTs with random areas of vertically aligned carbon nanotubes.

## Experimental

### Synthesis of graphene

Graphene was synthesized by chemical vapor deposition of methane (Air Liquide) on copper foil (Alfa Aesar, 99.8% purity, 0.025 mm thickness) at 1000 °C and 5 mbar in a hydrogen atmosphere (Air Liquide). After synthesis, the copper foil was rapidly removed from the heating zone of the setup under reduced pressure in a pure hydrogen atmosphere.

### Modified chemical etching

The backside of the copper foil was etched in oxygen plasma (Diener Electronics, Plasma System Femto, 300 W power limited to 200 W). The foil was etched with a gas flow of 12 mL/min oxygen (Air Liquide) at 30 °C and 0.8 mbar for 5 min using maximum power. Finally, a standard adhesive tape (Tesa tape) was attached to the edges of the copper foil (Figure S1, Supporting Information File 1). The etching chamber was constructed of two glued polystyrene petridishes (Carl Roth, 94 mm und 145 mm in diameter) with the inner compartment perforated (holes of 1.6 mm in diameter) at the base of the separation (Figure S2, Supporting Information File 1). A 1 M aqueous solution of ammonium persulfate was prepared by adding 1 L distilled water to 228.2 g of ammonium persulfate (Sigma-Aldrich, 98% purity). 190 mL of etchant was used to dissolve a 20 × 40 × 0.025 mm copper foil over a period of more than 72 h. A 1 M hydrochloric solution of iron(III) chloride was prepared by adding 700 mL water and 300 mL 37 wt % hydrochloric acid to 162.2 g iron(III) chloride (Alfa Aesar, anhydrous, 98% purity) on ice. The solution was stirred for 2 h and filtered. 190 mL of etchant was used to dissolve the 20 × 40 × 0.025 mm copper foil over a period of 3 h. Etchants of different concentrations were prepared by adjusting the ratio of water to hydrochloric acid and of iron(III) chloride to solvent.

### Post-processing by annealing

Graphene etched with a 1 M hydrochloric solution of iron(III) chloride was transferred onto a TEM sample support and analyzed. The TEM sample support was then placed in a tube furnace and annealed at 450 °C for 24 h in pure hydrogen (Air Liquide) at a flow rate of 10 mL/min.

### Synthesis of carbon nanotubes

Carbon nanotubes were synthesized using water-assisted chemical vapor deposition of ethene (Air Liquide) on SiO_2_/Si wafers (Silicon Materials) covered with an iron oxide nanoparticle/graphene composite at 875 °C in a hydrogen/argon atmosphere (Air Liquide). After synthesis, the setup was purged with argon and cooled to 450 °C for extraction of samples. All parameters were optimized for the synthesis of vertically aligned CNTs on SiO_2_/Si coated with aluminium and iron [45–47].

### Materials analysis

Graphene transferred onto SiO_2_/Si wafers was analyzed by Raman spectroscopy (Horiba, LabRAM HR8000, Lab Spec 5) at a wavelength of 514.5 nm. For analysis by TEM, graphene was transferred onto the TEM sample support (Electron Microscopy Sciences, lacey carbon film on 300 mesh copper grid) which was fixed to a SiO_2_/Si wafer during the transfer process by adhesive tape. The TEM was operated at an acceleration voltage of 200 keV (FEI, Tecnai G2F20@200keV, equipped with EDAX EDX detector). EDX and SAED spectra of the iron oxide nanoparticles were aquired simultaneously in the TEM. For high-resolution images, the TEM (FEI, Titan3 80-300 microscope with CS image corrector) was operated at 80 keV. Carbon nanotubes were analyzed using SEM (FEI, Philips XL30 FEG) with an acceleration voltage of 30 keV. Additional characterization of CNTs by TEM and Raman spectroscopy were found to be similar to the analysis of graphene.

## Supporting Information

The supporting information features additional figures regarding the modified transfer process, characterization of iron oxide nanoparticles, analysis of etching parameters and CNT growth.

File 1 Additional figures.
