# Supplementary material for: A systematic study of the controlled generation of crystalline iron oxide nanoparticles on graphene using a chemical etching process
Source: Beilstein J Nanotechnol. 2017 Sep 26;8:2017–25. doi: 10.3762/bjnano.8.202 (PMC5629405; doi:10.3762/bjnano.8.202)
Supplement: File 1 — Additional figures. [file Beilstein_J_Nanotechnol-08-2017-s001.pdf]

Supporting Information

for

A systematic study of the controlled generation of crystalline iron oxide nanoparticles on graphene using a chemical etching process

Peter Krauß, Jörg Engstler and Jörg J. Schneider*

Address: Fachbereich Chemie, Eduard-Zintl-Institut für Anorganische und Physikalische Chemie, Technische Universität Darmstadt, Alarich-Weiss Str. 12, 64287 Darmstadt, Germany

Email: Jörg J. Schneider* - joerg.schneider@ac.chemie.tu-darmstadt.de

* Corresponding author

Additional figures

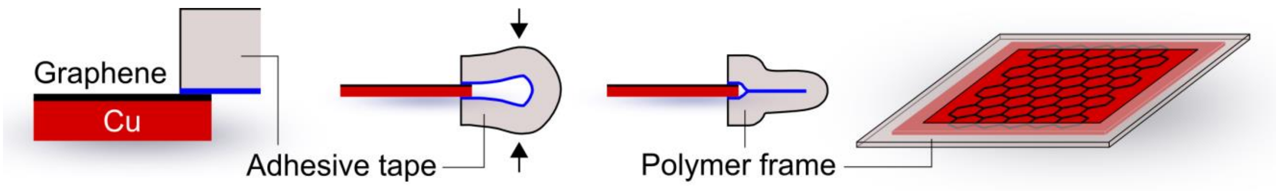

Figure S1: Attaching the encircling polymer frame to graphene on copper foil. The frame consists of four individual parts which overlap.

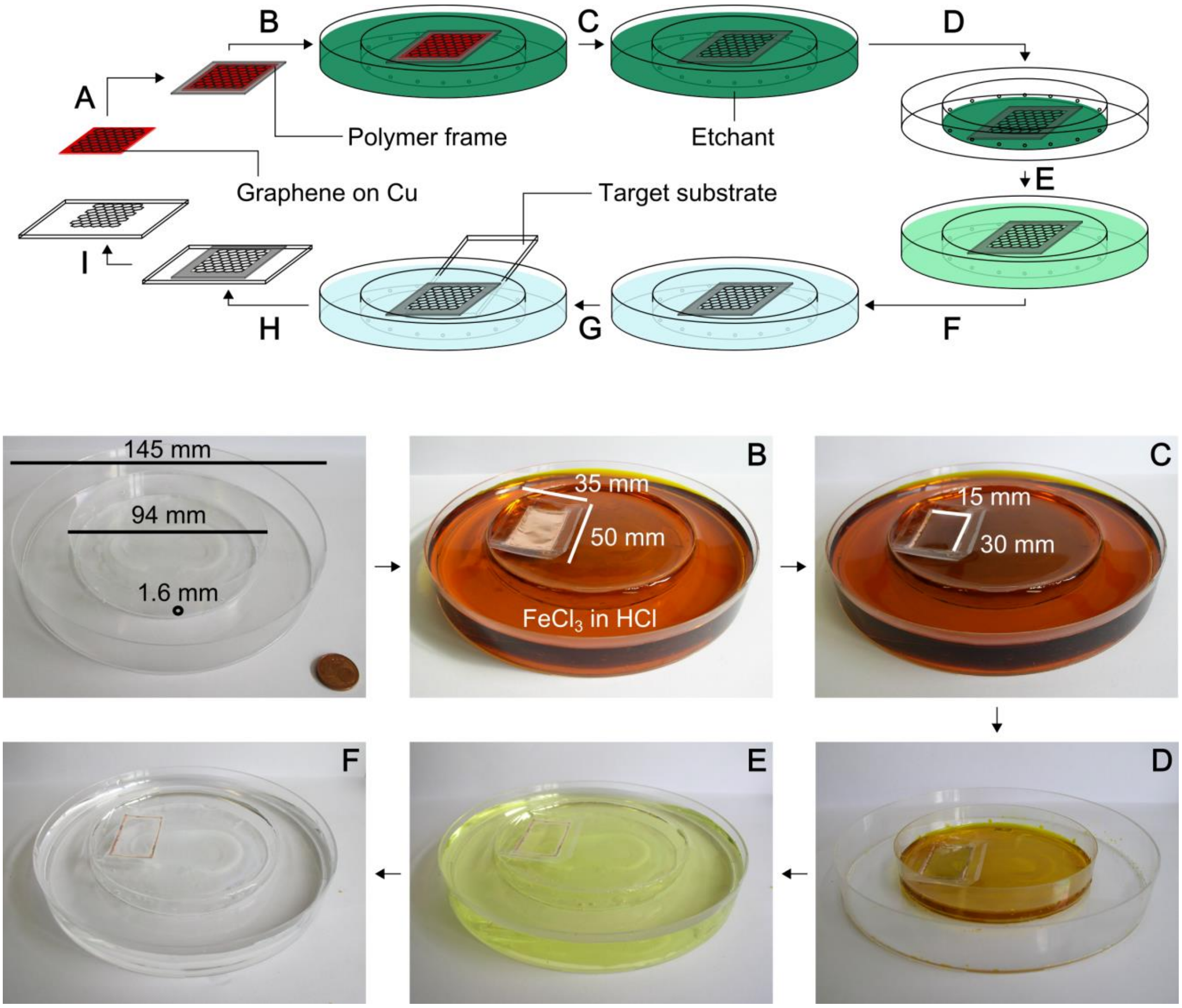

Figure S2: Modified chemical etching to transfer CVD graphene without polymer coating. (A) Adding an encircling polymer frame to graphene on catalyst foil. (B) Placing of supported graphene/catalyst foil on the etchant solution in the inner compartment of the subdivided etching chamber. The two compartments are

connected by small openings at the base of the inner compartment. (C) Etching of catalyst foil. (D) Draining of etchant from outer compartment. (E) Filling of outer compartment with water. (F) Repeating steps (D) – (E) several times for cleaning. (G) Scooping up the supported graphene with target substrate. (H) Drying of supported graphene. (I) Removing polymer support by lift-off and rinsing with water.

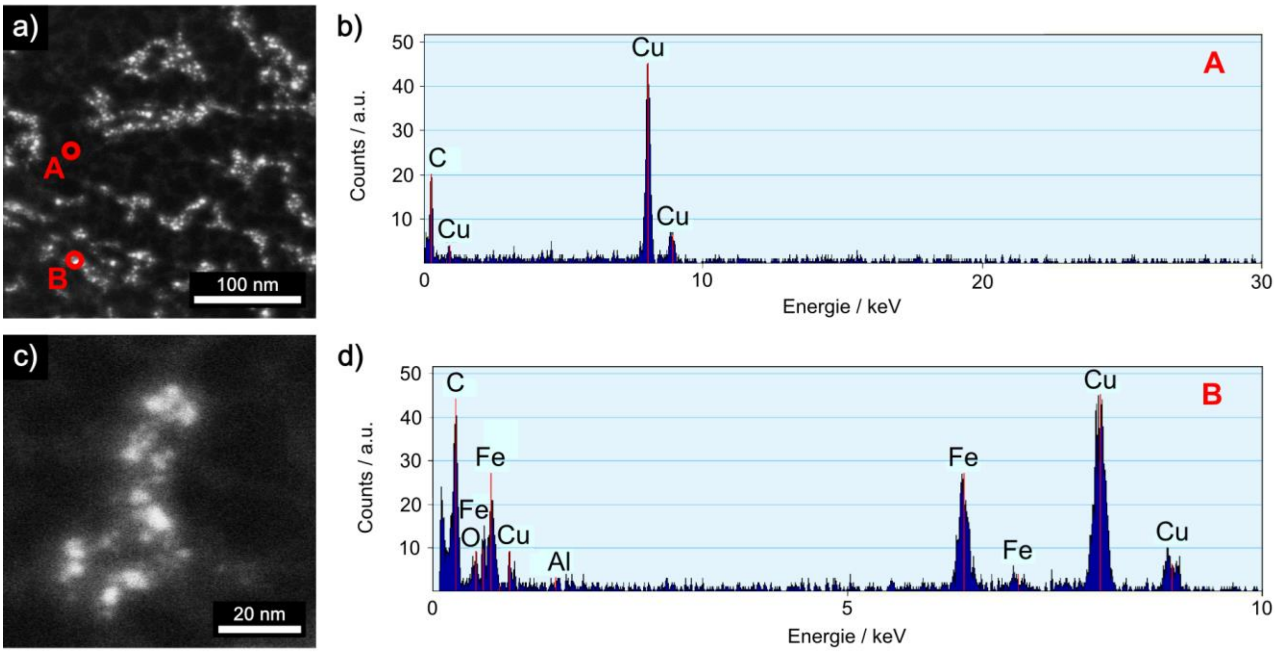

Figure S3: Characterization of iron oxide nanoparticles on CVD graphene and TEM sample support (lacey carbon film on copper grid) by high-angle annular dark-field imaging (HAADF) and energy-dispersive X-ray spectroscopy (EDX). a) and c) HAADF images at different magnification. b) EDX spectrum at position (A) in a) without nanoparticles. d) EDX spectrum at position (B) in a) with nanoparticles present. EDX data confirms the presence of carbon, oxygen, iron and copper (and aluminium, yet intensity is negligible). The results are in agreement with data from *Alemán* et al. and confirm the composition of the nanoparticles of iron and oxygen.

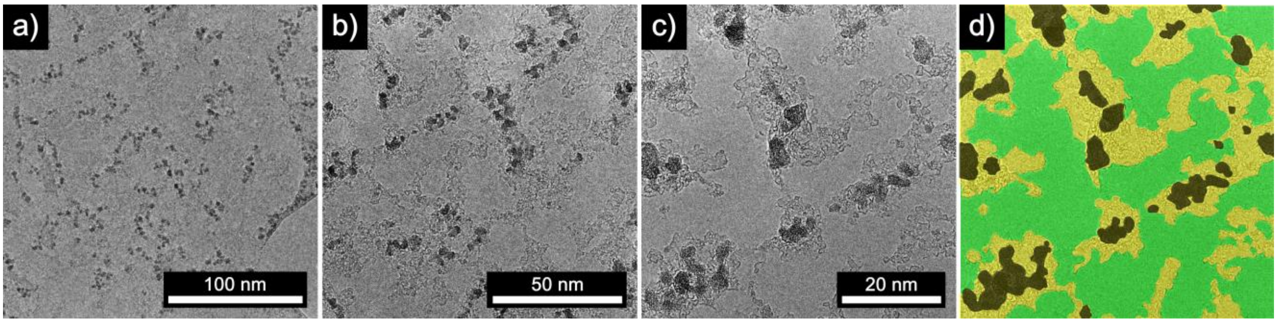

Figure S4: Iron oxide nanoparticles on graphene analysized by TEM. d) Artificially coloured image of c) with areas of monolayer graphene (green), few-layer graphene (yellow) and nanoparticles (black). Obviously nanoparticles tend to agglomerate in areas in which additional layers have formed, whereas no particles can be found on the continuous, pristine and flat monolayer in between.

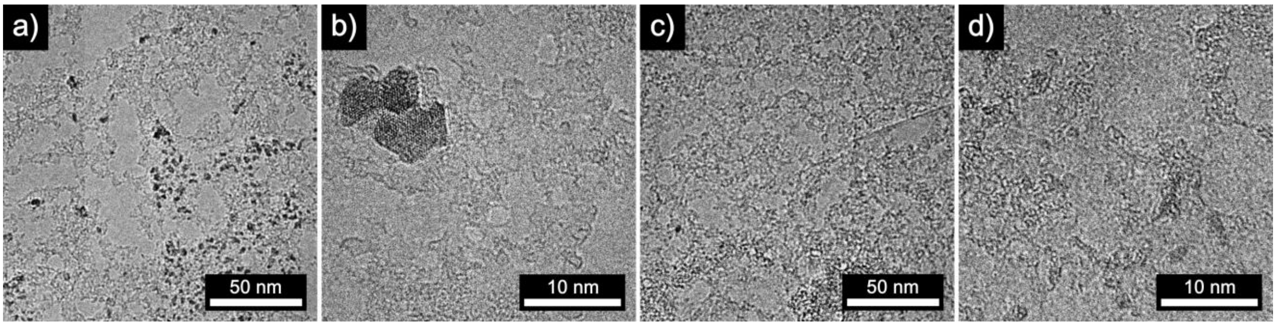

Figure S5: Images of iron oxide nanoparticles on graphene, synthesized using different concentrations of iron(III) chloride in 10 % hydrochloric acid. a) and b) 0.5 M $FeCl_3$. c) and d) 0.25 M $FeCl_3$. Reducing the concentration of iron(III) chloride to 0.25 M slightly decreases the average diameter of nanoparticles.

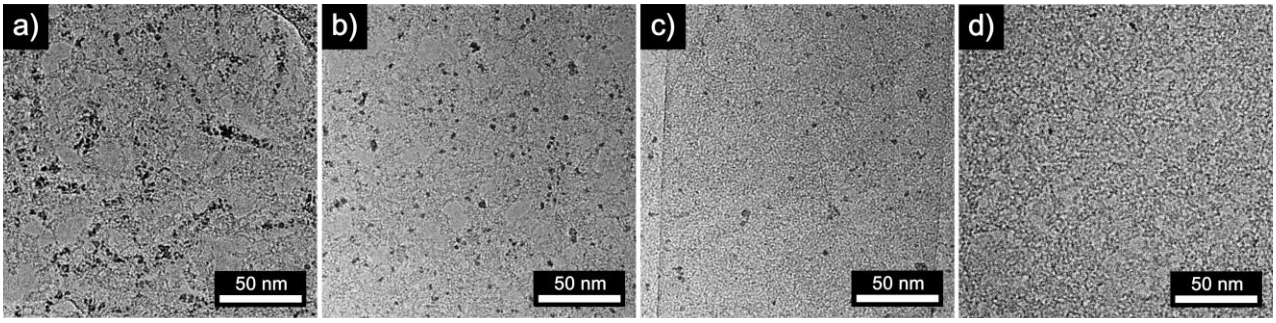

Figure S6: TEM micrographs of iron oxide nanoparticles on graphene using increased weight percents of hydrochloric acid as solvent for the etchant 1 M iron(III) chloride. a) 10 %. b) 20 %. c) 30 %. d) 37 %. Increasing the concentration of hydrochloric acid reduces the number of observed iron oxide nanoparticles drastically.

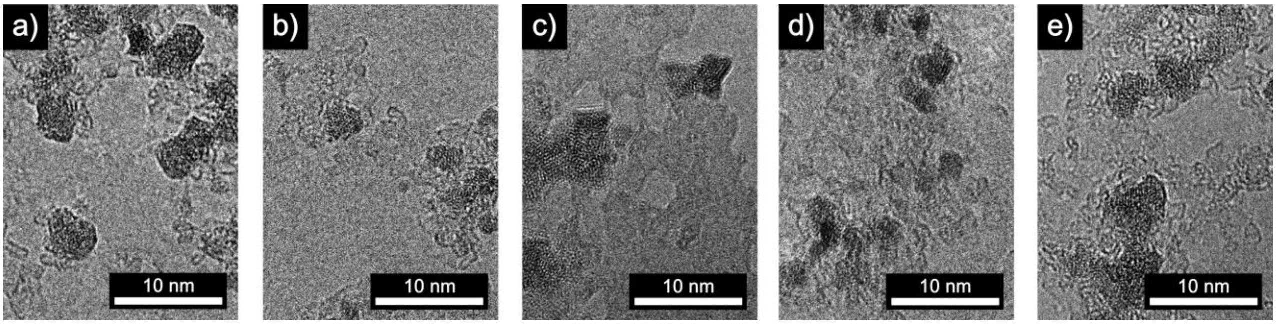

Figure S7: TEM images of iron oxide nanoparticles on CVD graphene after different periods of etching with a solution of 1 M iron(III) chloride in 10 % hydrochloric acid. a) 15 min (minimum time of etching referring to complete visual removal of copper). b) 30 min. c) 1 h. d) 3h. e) 1 d. Analysis by TEM indicates no variation in number, size or shape of the iron oxide nanoparticles.

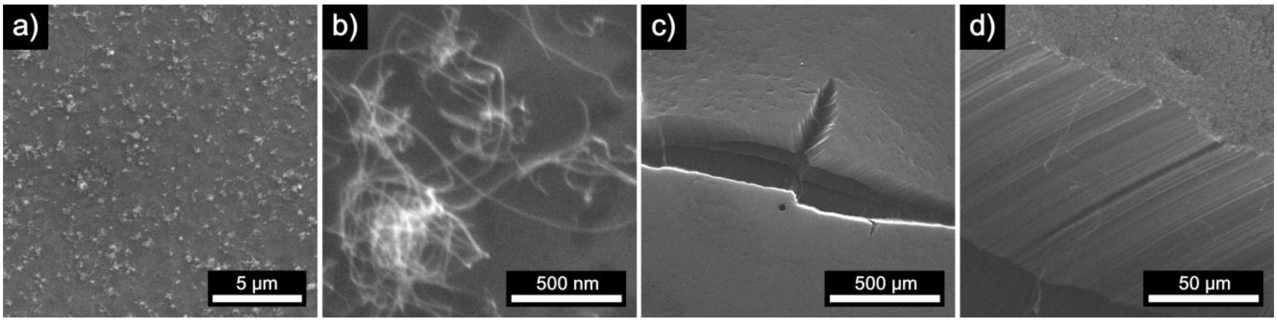

Figure S8: Characterization of CNTs grown on graphene, which was functionalized with iron oxide nanoparticles by modified etching process using a solution of 1 M iron(III) chloride in 10 % hydrochloric acid (comparable to Figure 2). a) and b) SEM images of irregularly grown CNTs. c) and d) On some samples areas of vertically aligned carbon nanotubes can also be detected.
